# Opening the door: midwives’ perceptions of two models of psychosocial assessment in pregnancy- a mixed methods study

**DOI:** 10.1186/s12884-020-03133-1

**Published:** 2020-08-07

**Authors:** V. Schmied., N. Reilly, E. Black, D. Kingston, K. Talcevska., V. Mule., M-P Austin

**Affiliations:** 1 School of Nursing & Midwifery, Western Sydney University, Locked Bag 1797 Penrith, Sydney, NSW 2751 Australia; 2 Research Centre for Generational Health and Ageing & School of Nursing and Midwifery, University of Newcastle, Newcastle, NSW Australia; 3 Perinatal and Women’s Mental Health Unit, St John of God Health Care and University of New South Wales, Sydney, Australia; 4 Drug and Alcohol Services, South Eastern Sydney Local Health District, 591 South Dowling Street, Surry Hills NSW 2010 and Discipline of Addiction Medicine, University of Sydney, Camperdown, NSW 2006 Australia; 5 School of Public Health and Community Medicine, Faculty of Medicine, University of NSW, Sydney, 2052 Australia; 6 Faculty of Nursing, University of Calgary, Calgary, Alberta Canada

**Keywords:** Risk assessment^, Psychosocial factors^, Pregnancy^, Midwives, Mental health, Mixed methods, ^mesh term.

## Abstract

**Background:**

One in five women experience psychological distress in the perinatal period. To support women appropriately, Australian guidelines recommend routine depression screening and psychosocial risk assessment by midwives in pregnancy. However, there is some evidence that current screening processes results in higher rates of false positives. The Perinatal Integrated Psychosocial Assessment (PIPA) Project compared two models of psychosocial assessment and referral – Usual Care and the PIPA model – with a view to improving referral decisions. This paper describes midwives’ perspectives on psychosocial assessment, depression screening and referral at the antenatal booking appointment and compares midwives’ experiences with, and perspectives on, the two models of care under investigation.

**Methods:**

A two-phase, convergent mixed methods design was used. Midwives providing antenatal care completed a self-report survey in phase one prior to implementation of the new model of psychosocial assessment (*n* = 26) and again in phase two, following implementation (*n* = 27). Sixteen midwives also participated in two focus groups in phase two. Quantitative and qualitative data were compared and integrated in the presentation of results and interpretation of findings.

**Results:**

Midwives supported psychosocial assessment believing it was a catalyst for ‘Opening the door” to conversations with women. Midwives were comfortable asking the questions and tailored their approach to build rapport and trust. Overall. midwives expressed favourable views towards the PIPA model. A greater proportion of midwives relied mostly or entirely on the suggested wording for the psychosocial questions in the PIPA model compared to Usual Care (44.4% vs 12.0%, *χ*^2^=5.17, *p*=.023, *φ* =-.36). All midwives reported finding the referral or action message displayed at the end of the PIPA psychosocial assessment to be ‘somewhat’ or ‘very’ helpful, compared to 42.3% in Usual Care (χ^2^ = 18.36, *p* < .001, *φ* = −.64). Midwives were also more likely to act on or implement the message often or all of the time) in the PIPA model (PIPA = 69.2% vs Usual Care = 32.0%, (χ^2^ = 5.66, *p* < .017, *φ* = −.37).

**Conclusion:**

The study identified benefits of the new model and can inform improvements in psychosocial screening, referral and related care processes within maternity settings. The study demonstrates that psychosocial assessment can, over time, become normalised and embedded in practice.

## Background

Mental health and social well-being in the perinatal period (from conception to one year following birth) is critical for the health of both mother and baby [1, 2]. A range of psychosocial stressors are associated with poorer maternal and infant outcomes, including recent life stress, poor partner relationship, lack of social support and a trauma history or past or current depression or anxiety [3]. To address the risks associated with these stressors, the Australian guidelines, “*Mental Health Care in the Perinatal Period: Australian Clinical Practice Guideline*” recommend depression screening and psychosocial risk assessment and monitoring throughout the perinatal period [4]. In New South Wales (NSW), such assessment has been mandated in the public health system since 2010 through the SAFE START policy [5], and has been conducted at the study site from 2000 onwards as part of the first antenatal visit conducted by midwives [6, 7]. Currently very few private hospitals with maternity services in NSW or Australia, routinely conduct psychosocial assessment [8].

While Australian [4] and to various degrees other guidelines [9], support assessment for psychosocial risk and depression in pregnancy, such an approach is not without controversy with ongoing concerns about potential over-pathologising or even inappropriate medicating of distressed but not clinically depressed women [10, 11]. A small Australian study conducted after the implementation of the SAFE START policy found that a high proportion of women (39%) met the criteria for psychosocial referral [12], while a clinical audit conducted at the study site found a significant rate of false positives under the Usual Care model. Such potential over-referral of women may also result in inadvertent over-servicing, reduced system efficacy, increased costs through service duplication, and increased anxiety for women being incorrectly identified as ‘at risk’ [12].

In response to these findings, the Perinatal Integrated Psychosocial Assessment (PIPA) Project was developed to provide greater evidence upon which to interpret the results of a woman’s psychosocial assessment; and base referral decisions. The PIPA Project compares two models of psychosocial assessment and referral as conducted by midwives at a large tertiary maternity hospital in Sydney, Australia. The methods of the PIPA Project are described in greater detail elsewhere [13].

Taking a mixed methods approach, the objectives of this paper are twofold. Firstly, to describe midwives’ perspectives on psychosocial assessment, depression screening, referral, related issues in the context of the antenatal booking appointment and follow up care; and secondly, to compare midwives’ perspectives and experiences on the two models of care under investigation (usual care and PIPA).

### Usual care

The SAFE START policy [5] provides a framework using a population health model for mothers, infants and their families. SAFE START involves universal psychosocial risk assessment and depression screening for all women as part of a comprehensive health assessment during both pregnancy and the postnatal periods. This assessment is linked to a network of supports and health-related services for those mothers, infants and families at risk of adverse physical and mental health outcomes. (5 p3)**.** The SAFE START framework includes a multidisciplinary case meeting. Referral pathways and support mechanisms vary between maternity services. The referral network offered at the study site is described below.

In this study, Usual Care refers to one component of the SAFE START policy and guidelines that is, the process of psychosocial assessment (including screening for domestic violence) and depression screening to identify need and referral. We have not investigated other components of the SAFE START framework. Usual Care consisted of nine largely closed-ended questions plus two questions around past episodes of mental health problems (if that item was endorsed); and the Edinburgh Postnatal Depression Scale (EPDS) [13]. The following areas associated with poorer maternal / infant outcomes were assessed: low level of social support; recent (past 12 month) stress; tendency to worry and level of self-confidence; mental health history; experience of abuse in childhood; previous contact with child protection services or having a dependent child in the care of another person; substance use, and recent experience of family (domestic) violence. Responses to these items and the EPDS determined the level of psychosocial risk as either level 1 (no endorsed risk factors); level 2 (reporting one or more social issues, recent major stressors, history of adverse childhood events and/or history of maternal or partner mental illness); or level 3 (reporting one or more complex risk factors such as homelessness, family violence and serious mental health issues). Upon completion, a flag indicated whether any psychosocial issues were identified. The flag recommended that women be referred for additional support or mental health assessment if clinically appropriate; i.e. the decision to refer was left open.

#### The PIPA model

The PIPA model, consisting of the Antenatal Risk Questionnaire-Revised (ANRQ-R) and EPDS, built on the Usual Care model with a more comprehensive assessment. The original Antenatal Risk Questionnaire [6] was revised (ANRQ-R) to include questions relating to substance use and domestic violence, as well as additional exploratory questions around past episodes of mental health problems (if the mental health item was endorsed). The algorithm then generated six levels of psychosocial risk (from no risk through to high or complex risk) based on a combination of individual responses and total scores on the EPDS and ANRQ-R, accompanied by tailored recommendations (prompts) for psychosocial referral to the available onsite services.

The PIPA model generates a psychosocial risk level based on a *cumulative* measure of the impact of psychosocial risk factors (measured as a total score on the ANRQ-R). It measures the *severity* of a number of individual risk factors (whereas Usual Care identifies only the presence or absence of these risk factors). To achieve this PIPA *auto-scores* the questionnaires, eliminating manual scoring errors arising in this context [14]. PIPA also includes additional structured questions to explore self-harm on the EPDS when question 10 is endorsed, and enables the midwife to document clinical concerns not elicited by the questionnaires, (e.g. the woman’s clinical presentation at interview). This ‘Clinician Concerns’ option duly recognises the importance of midwives’ clinical acumen and provides an avenue for clinical review of that woman’s presenting issues and possible referral for psychosocial support irrespective of her questionnaire scores. A table outlining the differences between the two models is in Supplementary File 1.

#### Referral pathways

The same psychosocial referral pathways were available in both conditions. Options included: no referral; referral to psychoeducational groups; midwife to monitor by repeating the EPDS next visit; or, for women with more complex presentations, referral for review and support as appropriate by the multidisciplinary psychosocial care team. This team included representatives from social work, substance use, and Perinatal Mental Health services. Women with more complex presentations would also be referred on for case discussion at a weekly Multidisciplinary Case Discussion (MCD) meeting attended by a larger number and range of representatives from the available onsite psychosocial services.

Prior to the introduction of the PIPA model, midwives received formal training in the new assessment model facilitated by the last author. This comprised of a single 1 h in-service session. This was in addition to the informal individual psychosocial assessment training provided to new midwives when they start work at the study site.

## Methods

### Study design

**Fig. 1:**
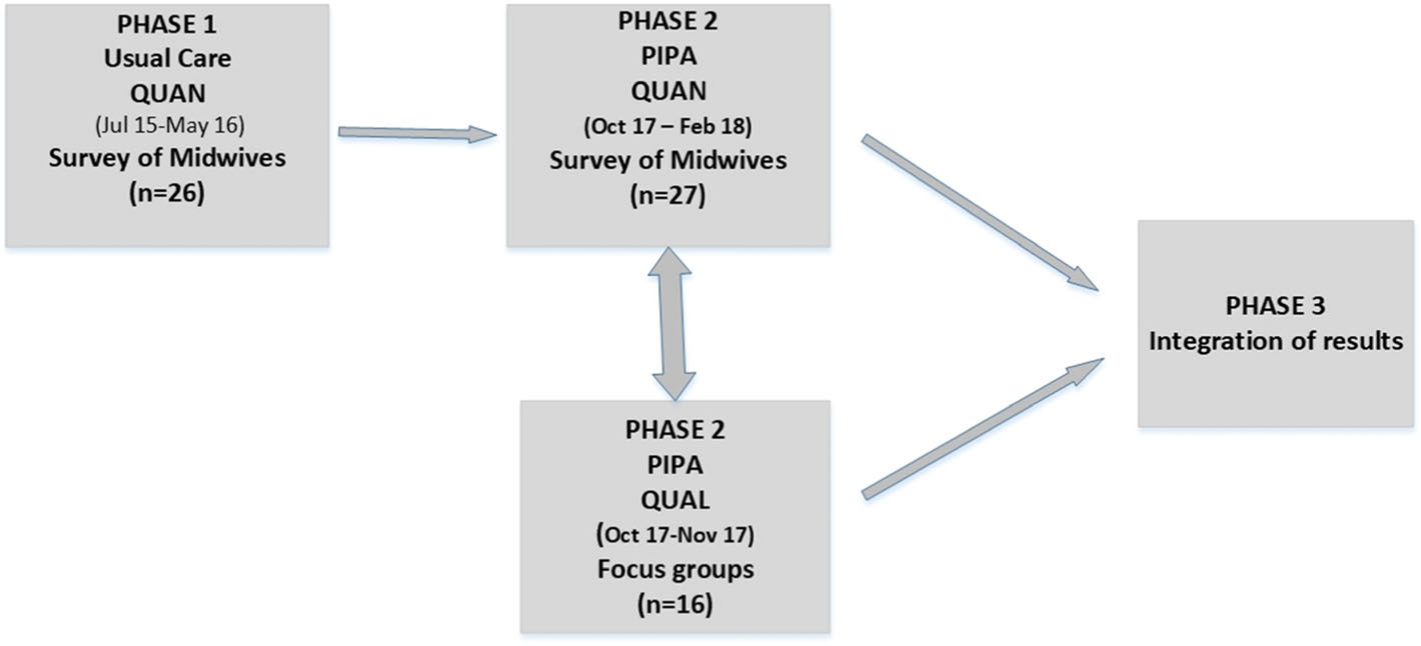
Mixed Methods Design

A two-phase, convergent mixed methods design was selected as the best approach to develop a more complete understanding of midwives’ experiences of psychosocial assessment and depression screening and their perceptions of the two models of care (Usual Care & PIPA) [15]. To make this comparison between the two models, midwives undertaking psychosocial assessment in phase one (Usual Care) were invited to complete a survey about their experiences with psychosocial assessment and depression screening and their perceptions of the Usual Care model. Approximately two years later in phase two of the study, midwives completed the same survey about the PIPA model with five additional questions on the PIPA condition and how it differed from the Usual Care model. At the same time in phase two, they were invited to participate in a focus group. We did not conduct a focus group in phase one as staff had recently provided their views in another study by the team (l7). However, the analysis of phase one survey data informed phase two focus group questions. Quantitative and qualitative data in phase two were then collected and analysed separately and integration of the quantitative and qualitative findings occurred when the results were compared and discussed. Our goal was to triangulate the findings from quantitative survey data and the qualitative focus group data. (see Fig. 1).

#### Ethics

The project was approved by the appropriate South East Sydney Local Health District Human Research Ethics Committee, approval number 14/117, 23 October 2014.

#### The study site

The study was conducted in a large tertiary public maternity hospital based in metropolitan Sydney NSW Australia with approximately 4000 births each year ( [16]). At this site a range of services and supports were available for women depending on their needs. This included referral to psychoeducational support groups for women who had experienced childhood abuse; social work and specialist services for drug and alcohol and or domestic violence; psychiatric and psychological services for counselling treatment and medication. The site was selected because the research team, in particular the last author, had a well-established relationship with the services and it offered the best place for the pilot study of the PIPA questions. This engagement was critical because the integration of the PIPA questions into the e-maternity system required considerable support from the IT department.

#### Participants and recruitment

The participants of this study were midwives undertaking antenatal psychosocial assessments either through the antenatal clinic or through the midwifery group practice (MGP) model [17]. The total number of midwives working in the antenatal clinic at midwifery group practice at both survey times points was approximately 60 (40 midwives in the clinic and 20 midwives in MGP). Midwives were recruited using a number of strategies. Following a briefing from the research team, managers distributed information flyers to midwives via email as well as posting information on the notice boards in the staff tea room and meeting rooms. The research team attended several staff meetings to answer questions and invite midwives to complete the survey and in phase two, to also participate in focus group discussions.

#### Survey design

The survey was developed specifically for the study and included sociodemographic and work history items, 18 likert-style questions that addressed individual aspects of the psychosocial assessment process, and two open ended/comments. The likert responses were on a 5-point scale (disagree strongly, disagree, neutral, agree, strongly agree). The additional items added to the PIPA condition consisted of three items regarding unique aspects of the PIPA model, and two directly comparing the two models. The survey questions can be viewed in the tables presenting results in the paper. The survey was pilot tested with two academic midwives and two clinical midwives. The purpose of the pilot was to confirm user-friendliness, clarity and acceptability (e.g. on length, content). It received positive feedback, with minor wording changes for clarity made. The second survey was the same as the first PLUS some additional PIPA questions. We did not pilot test the second survey.

#### Procedure

The surveys were distributed to midwives working in the antenatal clinics and in Midwifery Group Practice. Each paper based survey also included a coffee voucher attached as a thank you for participation. Midwives placed non-identifiable completed surveys in a box on the ward. The first survey (Usual Care) was conducted between August and November 2015 and the second survey was conducted between October 17 and February 18 (PIPA).

#### Focus groups

All midwives undertaking antenatal booking visits during the PIPA phase were invited to participate in a focus group lasting 45–60 min. Sixteen midwives attended one of two focus groups. A semi-structured focus group interview guide was prepared by VS in consultation with co-authors. It comprises 10 open ended questions. Questions were based around the study aims and were cross--referenced against the survey questions for midwives. Discussions were audio recorded with midwives’ consent and transcribed for analysis. Focus group questions can be found in the supplementary file 2.

#### Data analysis

Quantitative data from the two surveys were entered into the Statistical Package for the Social Sciences (SPSS) versions 24 [18], and analysed using descriptive and inferential statistics to address the two research aims. Missing survey data was left vacant and response options for some questions were collapsed. Responses to all phase one and phase two survey questions completed by midwives were compared using chi-square with effect size reported as Phi Coefficient (small effect = 0.1, medium effect = 0.3, large effect = 0.5).

The qualitative data from the focus groups were analysed using thematic analyses (Braun and Clarke’s, 2006) a six-step process of qualitative data analysis [19]. Initially EB, KT and VS become familiar with the data through repeated readings of all transcripts. Individually the researchers identified ideas and patterns in the data and proposed data codes using NVivo v11. These codes/ideas were then discussed with the full research team and a consensus formed. These codes and data exemplars were cross referenced with survey questions and responses and with further re-reading of the transcribed focus group data, linkages across the codes and preliminary themes were identified and then compared again with the survey responses. Exemplar quotes from the focus groups are presented; however, the focus group identifier is not used to ensure confidentiality.

#### **I**ntegration

Integration refers to the points in the study where ‘mixing’ of qualitative and quantitative methods occurs [20, 21]. Integration of data or findings from the qualitative and quantitative components of a study is a core characteristic of mixed methods research [22]. In this study integration occurred during the design, specifically planning the survey questions for phase one and two; and in relation to comparison of findings from analyses of both datasets, interpretation and discussion phases of the study (see Fig. 1). Each dataset was analysed separately and then examined for thematic patterns. Survey data and the focus group have been integrated in the thematic analysis and presentation of the results.

## Results

In total 26 midwives completed the first survey about the Usual Care assessment and 27 midwives did the second survey related to the PIPA assessment approach and comparing this to Usual Care. This represents around *two thirds of midwifery staff* undertaking booking visits. Nine midwives completed *both* the Usual Care and PIPA surveys. Sixteen midwives participated in the focus groups. Characteristics of the samples are shown in Table 1.

**Table 1:** Characteristics of survey participants (midwives)

	Usual Care surveys *N* = 26	PIPA Condition surveys *N* = 27
Midwife age (years)		
20–29	6 (24%)	4 (15%)
30–39	2 (8%)	8 (31%)
40–49	8 (32%)	6 (23%)
50–59	9 (36%)	8 (31%)
Mean years experience (SD, range)	14.4 (9.13, 0.8–30)	13.6 (9.65, 0.6–32)
Mean years experience conducting psychosocial assessments (SD, range)	7.6 (5.55, 0.4–20)	9.4 (7.49, 0.8–24)
Mean booking visits conducted/week	3.3 (3.02, 0–11) ^c^	2.7 (2.51, 0.5–10)
N providing care under		
standard models (e.g. GP Shared Care^a^)	12 (48%)^d^	9 (33%)
Midwifery Group Practice^b^	13 (52%)	17 (63%)
Other (manager)	–	1 (4%)
N completed formal training in psychosocial assessment^d^		
in past year	4 (17%)	13 (48%)
1+ year ago	13 (54%)	10 (37%)
Never	7 (29%)	3 (11%)
N completed training in the PIPA condition	n/a	24 89%)^e^

^a^Shared care is an arrangement between a birthing hospital or other birth setting and a general practitioner (GP). Women will see their GP for some pregnancy appointments, and also have hospital appointments in early and later pregnancy

^b^Midwifery Group Practice (MGP) is a model of maternity care where women receive one to one care with a midwife for their pregnancy, labour, birth and postnatal care

^c^ one midwife recorded ‘0’ for bookings per week because at the time of the survey she was not undertaking booking visits; however, due to her extensive experience, her comments were retained in analyses

^d^*n* = 1 case missing data

^e^*n* = 2 cases missing data in UC and PIPA conditions, respectively

There were no significant differences between survey groups in terms of years of midwifery experience nor experience conducting psychosocial assessments between respondents. Similarly, no difference was detected between these groups in regard to the numbers of first appointments or booking visits – at which psychosocial assessments are typically conducted – being done per week. Midwives who participated in the focus group had between 1 and 25 years’ experience as a midwife with a mean of 15 years and worked in a range of roles across the spectrum of midwifery care. They had been providing antenatal care at the study site for between 1.5 and 25 years with a mean of 14 years.

### Overview of survey results

Results showed statistically significant differences in three of the 16 survey questions. A greater proportion of midwives reported relying mostly or entirely on the suggested wording for the psychosocial questions in the PIPA model compared to Usual Care (44.4% vs 12.0%, *χ*^2^ = 5.17, *p* = .023, *φ* = −.36) (see Table 2). All midwives reported finding the referral or action message displayed at the end of the PIPA psychosocial assessment to be ‘somewhat’ or ‘very’ helpful, compared to 42.3% in Usual Care (*χ*^2^ = 18.36, *p* < .001, *φ* = −.64). This message was also more likely to be followed often or all of the time in the PIPA model (PIPA = 69.2% vs Usual Care =32.0%, (*χ*^2^ = 5.66, *p* < .017, *φ* = −.37) (see Table 2).

**Table 2:** Midwives’ practices and comfort in undertaking psychosocial assessment

	Usual Care	PIPA condition	Result
**Q1. Comfort with discussing psychosocial issues with pregnant women**	*N* = 25	*n* = 27	
Very/somewhat comfortable	24 (96%)	27 (93%)	ns
Very/somewhat uncomfortable	1 (4%)	2 (7%)	
**Q2. Reliance on programmed wording vs. own wording**	*N* = 25	*n* = 27	
Entirely/mostly rely on programmed wording	3 (12%)	12 (44%)	χ2 = 5.17, *p* = .023, φ = −.36
Entirely rely on own wording	22 (88%)	15 (56%)	
**Q3. Comfort using the questions that appear onscreen**	*N* = 26	*n* = 26	
Very/somewhat comfortable	23 (89%)	26 (100%)	ns
Somewhat/Very uncomfortable	3 (12%)^a^	0	
**Q4. Appropriateness of psychosocial screen at booking rather than subsequent visit**	*N* = 26	*n* = 27	
Very/somewhat appropriate	23 (89%)	27 (100%)	ns
Somewhat/Very inappropriate	3 (12%)^a^	0	
**Q5. Ease with which psychosocial assessment could be completed on computer**	*N* = 26	*n* = 27	
Very/somewhat easy	26 (100%)	27 (100%)	ns
Somewhat/Very difficult	0	0	
**Q6. Clarity with which the psychosocial questions identify key psychosocial issues**	*N* = 26	*n* = 26	
Very/somewhat clearly	22 (85%)	26 (100%)	ns
Not very clearly	4 (15%)	0	
**Q7. Comfort providing feedback from psychosocial assessment to women with more complex issues (e..g DV, substance use, child protection services, serious mental health problems)**	*N* = 26	*n* = 27	
Very/somewhat comfortable	22 (85%)	22 (82%)	ns
Somewhat/Very uncomfortable	4 (15%)	5 (19%)^a^	
**Q8. Confidence in discussing referral options for women with complex issues**	*N* = 26	*n* = 26	
Very/somewhat confident	21 (81%)	24 (92%)	ns
Not very confident	5 (19%)	2 (8%)	
**Q9. How often EPDS results disussed with women in your care**			
**Total elevated score**	*N* = 26	*N* = 27	
Most or all of the time	25 (96%)	23 (85%)	ns
Some of the time	1 (4%)	3 (11%)	
Hardly ever or never	0	1 (4%)	
**When score is not elevated**	*N* = 26	*N* = 27	
Most or all of the time	16 (62%)	19 (70%)	ns
Some of the time	9 (35%)	6 (22%)	
Hardly ever or never	1 (4%)^a^	2 (7%)^a^	
**Positive Q10 (thoughts of self harm)**	*N* = 25	*N* = 26	
Most or all of the time	24 (96%)	23 (89%)	ns
Some of the time	1 (4%)	1 (4%)	
Hardly ever or never	0	2 (8%)^a^	
**Q10. Confidence in discussing positive responses to Q10 (thoughts of self harm)**	*N* = 26	*N* = 25	
Very/somewhat confident	21 (81%)	21 (84%)	ns
Not very confident/Not confident at all	5 (19%)	4 (16%)	
**Q11. Helpfulness of onscreen action prompt/s**	*N* = 26	*N* = 26	
Very/somewhat helpful	11 (42%)	26 (100%)	χ2 = 18.36, *p* < .001, φ = −.64
Somewhat/Very unhelpful	15 (58%)	0	
**Q12. How often do you follow these messages?**	*N* = 25	*N* = 26	
Most of the time	8 (32%)	18 (69%)	χ2 = 5.66, *p* < .017, φ = −.37
Some of the time/Hardly ever or never	17 (68%)	8 (31%)	
**Q 13. In general, does the booking in time allocation allow for adequate psychosocial assessment?**	*N* = 26	*n* = 26	
Yes there is more than enough time/ Yes there is usually the right amount of time	14 (54%)	14 (54%)	ns
Yes but there is barely enough time/No usually there isn’t enough time	12 (46%)	12 (46%)	ns
**Q 14. How clinically useful do you find the Psychosocial Summary Report?**	*N* = 24	*N* = 27	
Very/somewhat useful	21 (88%)	24 (89%)	ns
Not very useful/Not useful at all	3 (13%)^a^	3 (11%)	
**Q15. At subsequent visits, how often do you refer to the Psychosocial Summary Reports that is [generated] at booking?**	*N* = 24	*N* = 26	
Most of the time	7 (29%)	7 (27%)	ns
Some of the time	12 (50%)	16 (62%)	
Hardly ever or never	5 (21%)	3 (12%)^a^	

*Does not total 100% due to rounding

### Overview of focus group findings

Thematic analysis of the focus groups revealed five main themes: ‘Opening the door: Benefits for Women’, ‘It’s become second nature: comfort in asking the questions’; ‘Tailoring my approach to build rapport and trust’; *‘But it’s a rush at times’* and; *‘PIPA is more in-depth and relevant’ but …*’. The themes are presented in Table 3. below and demonstrate the integration of survey and focus group data.

**Table 3:** Summary of integrated themes (survey & focus groups)

Theme number	Themes	Sub Themes	QUANT survey data	QUAL focus groups
1	“Opening the door”: benefits for women			•
2	“It’s become second nature”: comfortable asking the questions		•	•
3	Tailoring my approach to build rapport and trust	Setting the scene		•
		Adapting the questions	•	•
		Administration mode: midwife vs computer	•	•
*4*	*‘But it’s a rush at times’*		•	•
*5*	*‘PIPA is more in-depth and relevant’*	*The right questions*	•	•
		*Scoring risk*	•	•
		*Tailored action prompts*	•	•
		*The clinician’s concerns box: the importance of clinical judgement*	•	•

### Theme 1. ‘Opening the door’: benefits for women

The midwives believed that psychosocial assessment and depression screening was an important part of their practice with potential benefits for women. In the focus groups, some midwives described this as ‘opening the door’, offering the opportunity for women to have their concerns heard and validated. However, participants also acknowledged that the questions they were asking were sensitive. One midwife described the benefits in the following way:

*it’s helpful for … (women) to be asked questions that are explicit about their history so that they start to reflect on things that maybe have happened and that they haven’t really thought about it …*. *and it can be an impetus for change so I think that making it a normal part of the conversation around maternity can be very helpful.*

In the survey, almost all respondents in both models indicated it was appropriate to conduct psychosocial assessment at the first consultation and booking visit (Table 2, Q4). This was elaborated on in the focus groups. Even when women were not comfortable to discuss issues at the first visit, midwives viewed these questions as an important first step. Several midwives viewed the psychosocial assessment as the start of an ongoing conversation, noting that, *“as you get to know women better, sometimes they start to open up more, expand on things”*. Another added,

“*if … they’re not opening up, … they’ll go home and think about it and reflect on that and they may come back the next time and open up a bit more. It’s just opening the door, isn’t it?”*

At the same time, midwives recognised the challenges of psychosocial assessment noting that the information gathered was sensitive information and that some women are concerned about how their personal information is used. They cited examples of women’s concerns around access to and sharing of mental health history, childhood experiences of abuse and recent/current domestic violence. For example, although hospital protocols direct staff to inform women of the limits of confidentiality, one participant provided the following example:

*She (the woman) disclosed (childhood experiences). At the end of the conversation, I said, “We’ll be talking to the GP. They’ll be across this.” She was really upset, because it’s her family GP. So I think we need to be careful and ask the woman whether or not you mind this being shared with your GP.*

### Theme 2. “It’s become second nature”, but it is complex

Almost all participants indicated that they were comfortable or very comfortable asking women the psychosocial questions and that they were comfortable to ask the questions that appeared on the screen (see Table 2, Q1,3). In the focus groups midwives also reported generally feeling comfortable administering the psychosocial questions. They described psychosocial assessment as a skill that had become ‘second nature’, saying that, “*I thought it might have been quite difficult [to ask women the questions]. As you go along, you just realise it’s part of the whole process, looking at them holistically”.* Another added, *“I think the more comfortable you seem with it, the more comfortable they seem with it. If you seem uncomfortable then you’re introducing this idea that it’s something that needs to be shameful and embarrassing’.*

In contrast, one midwife indicated that it was very rare that she would ask the psychosocial questions at a first or subsequent visit, because she worked with a vulnerable population, saying, “*They’re just not appropriate questions to ask”.*

The majority of survey participants indicated that they were comfortable with providing feedback from psychosocial assessment to women with more complex issues (e.g. domestic/intimate partner violence, substance use, serious mental health problems) (Table 2, Q7).

In regard to the EPDS, midwives found value in reviewing women’s individual responses to the EPDS as well as the total score, regardless of whether the score was elevated or not, and thoughts of self-harm were typically (but not always) explored (Table 2, Q9). Midwives stated that responses to the EPDS gave important information but this needed to be considered within the context of women’s responses to the broader psychosocial questions. For example, one midwife suggested that a raised score on the EPDS may reflect pregnancy symptoms, saying “it’s because she’s vomiting every half hour” and “not because she has depression”.

Midwives’ confidence discussing thoughts of self-harm varied, but most reported themselves to be ‘very’ or ‘somewhat’ confident. The pre-programmed exploration prompts included in the PIPA model related to self-harm were found to be helpful (see Table 4, Q1) but a third of midwives had not administered these items due to low endorsement of EPDS Question 10 (risk of self-harm).

**Table 4:** Midwives perceptions of additional features in the PIPA model

**Q1. How useful are the Q10 exploration prompts?**	*N* = 27
Very useful	13 (48%)
Somewhat useful	5 (19%)
Not very useful/Not at all useful	0
Not applicable- I haven’t used them yet	9 (33%)
**Q2. How useful is the woman’s psychosocial risk level?**	*n* = 26
Very useful	11 (42%)
Somewhat useful	11 (42%)
Not very useful/Not at all useful	0
I haven’t seen this come up	4 (15%)
**Q3. Generally, how helpful is the PIPA [model] in eMaternity in assisting you to establish clear referral pathways?**	*n* = 26
Very helpful	16 (62%)
Somewhat helpful	10 (39%)^a^
Somewhat unhelpful/Very unhelpful	0
**Q4. Which psychosocial [model] have you found more helpful when deciding level of psychosocial risk?**	*n* = 25
They are both about the same	8 (32%)
I find the usual folder easier to use	4 (16%)
I find the PIPA folder easier to use	13 (52%)
**… when and where to refer women in your care?**	
They are both about the same	5 (20%)
I find the usual folder easier to use	5 (20%)
I find the PIPA folder easier to use	15 (60%)

^a^Does not total 100% due to rounding

### Theme 3. Tailoring my approach to build rapport and trust

To address the sensitive nature of the screening and assessment, midwives described a number of practices captured in these themes: ‘setting the scene’; ‘adapting the questions’; and, ‘mode of administration; midwife vs. computer”.

#### Sub theme 3.1: setting the scene

While the midwives were comfortable conducting psychological assessment, they recognised that questions could often be confronting for women. In the focus groups they discussed the importance of being upfront with and sensitive towards women, building rapport prior to and during assessment and being supportive of women during the assessment process. The midwives reflected on the importance of clearly explaining the content and purpose of the screening questions, stating that they explained the purpose to women, *“to help her see the significance, the relevance for the questions.”*. The following quote highlights that setting the scene allows the women to open up: “*Once women understand why we’re asking the questions, they’re much more open to sharing their experiences as well rather than being quite guarded”.*

#### Sub theme 3.2: adapting the questions

Overall, midwives relied mainly on their own wording in both the Usual Care and PIPA models, although there was a significant decrease in this practice in the PIPA model. In the Usual Care model only 12% of respondents indicated that they relied on the pre-programmed wording of questions while 88% relied mainly on their own wording. In comparison, in the PIPA model 44% relied entirely on the pre-programmed wording of questions (*χ*^2^ = 5.17, *p* = .023, *φ* = −.36) (see Table 2, Q2).

In the focus groups midwives explained that there were some questions that women had difficulty understanding, particularly women from culturally and linguistically diverse backgrounds. For example, one midwife indicated, *‘the (EPDS) question, things have been getting on top of me and the question on harming myself, I also have to explain that a lot.‘* When women did not understand the question or seemed perplexed by the question, midwives indicated that they try ‘*other ways’* to approach the subject.

#### *Sub theme 3.3:* mode of administration: midwife vs. computer

Midwives had different approaches to conducting the psychosocial assessment. All participants in both Usual Care and PIPA models reported that it was either very or somewhat easy to complete psychosocial assessment on computer (Table 2, Q5). However, in the focus groups midwives raised some concerns that while entering information on the computer they may miss seeing a woman’s response to the question asked, “*… looking at the computer. I get to look at their face less. I feel like (I) might miss signs. Sometimes you say, “How are you?“ And then just their eye might just... tear up. And you might have missed that sign, because you’re looking at the stupid computer”.*

The quality of information was considered by some midwives to be better if completed by the midwife and women together, enabling clarification of questions and responses as required, and facilitating rapport, particularly in the context of sensitive or unexpected questions. One said, “*I think it’s better … to go through it together rather than just sitting in front of a screen … I mean, sometimes you just get a sense from people. Sometimes they come in and they’re flat in affect and they just seem like there’s something in their past, or something going on”.* Another added, *“I usually sit with the computer facing them as well. Just so that they can see the questions that it’s on the computer...”.*

Other midwives preferred to ask the women to complete both questionnaires by themselves, or the EPDS only. This could be to save time, or to improve honesty and accuracy of responses. Midwives commented, “*I get them to self-complete and then I go through it with them afterwards and discuss it and ask any further questions … on certain issues. In the clinical concerns [free text box], I’ll flesh out what conversation we had based on anything that was flagged”.* Another added, *“I find that their responses are less filtered when they’re just directly typing into a computer rather than having to look someone in the eye. When you’re making decisions about what you do and don’t disclose”.*

Exceptions included some women from culturally and linguistically diverse backgrounds, where it could be best completed with, rather than by, the woman.

### Theme 4: ‘But it is a rush at times’

Time was a key theme arising from the analysis and this was equally an issue for both Usual Care and PIPA. Thus, 46% of respondents in both conditions indicated that they had barely enough time to complete the assessment adequately (Table 2, Q13). Time restrictions were also emphasised in the focus groups, where midwives explained that they felt like they were just going through question after question and this impacted on the rapport they developed with women. This was particularly the case in the PIPA condition, which was implemented alongside a new and more comprehensive but time consuming antenatal intake assessment. One midwife commented, “it is a rush at times; and another stated. “*..talking (with women) about their life a little bit as you go, but I find in the PIPA assessment there’s less time to do that. I find it’s more questions, to plough through. I find I don’t build the same rapport as in usual care”.*

### Theme 5. ‘PIPA is more in-depth and relevant’: comparing the two models

The change from Usual Care to PIPA (and its scored ANRQ-R) brought new processes, new or additional questions, inclusion of decision-aid support and more nuanced referral pathways as well as a fully computerised system as noted above. Based on these changes, midwives were asked, in the survey, to rate or comment the structure of the questions, the generation of PIPA psychosocial risk levels and scores and the revised referral pathways and more generally compare Usual Care to PIPA models. Midwives also discussed the two models in the focus group discussions.

### Sub theme 5.1: the right questions: opening up relevant dialogue

In the survey, the majority of midwives reported that the questions used in Usual Care and in the PIPA model were clear (see Table 2, Q6). However, in the focus group discussion, midwives described the PIPA questions as, “*More in depth. It’s (PIPA) more relevant.”* and “*It (PIPA) asks them more specific (questions), whereas the other one (*Usual Care) *was a bit vague”.* This enabled the midwife to formulate a picture of each woman’s unique situation, strengths, supports and challenges:

*It’s like anything, with good structure gives better results … you could be so busy 1 day that you’ve [missed something], because if [the screening tool is] wishy washy it allows you to do that. But if you’ve got a thorough, clean-cut [screening tool]... you got to ask this, this is all part of it, then it shouldn’t get missed.*

Midwives also particularly liked the addition in PIPA of a new question related to the woman’s relationship with her own mother and her childhood experiences. One midwife commented, “*Well I think the question about, “When you were growing up, was your mother emotionally supportive of you?“ Which is in the ANRQ, I think it gets women to think;. they’re going to be a mother … how were they mothered?”*

Despite the overall preference for the PIPA model and the use of the ANRQ-R, midwives felt that the question *“do you think having this baby is going to change your relationship with your partner?” -*included previously in the Usual Care model- should have been retained in the PIPA questions, saying that, “*Some people don’t think about it at all. … I certainly over the years had people come in and say, “This baby’s going to save my marriage”.*

#### Sub theme 5.2: value of PIPA generated psychosocial ‘risk level’ and ANRQ-R total scores

The PIPA model provided midwives with immediate feedback as to whether the woman was considered high, medium or low psychosocial risk. Eleven respondents indicated they found this psychosocial risk level useful; another 11 midwives indicated it was somewhat useful. (Table 4, Q2). Focus group participants also indicated that the risk level was of no particular benefit in their practice and that they tended to put the case file “*in the box”* (method by which midwives could refer women on to the MCD meeting for review) for all women with risk identified, regardless of the pathway recommended.

Opinions on the clinical value of the total ANRQ-R score (as a measure of number of risk factors) were mixed. On the one hand, midwives commented that the combination of the ANRQ-R and EPDS scores in the PIPA model enabled them to obtain a broader contextual understanding of women’s situations. One midwife reported, “*I like the ANRQ-R. Much better than just doing the Edinburgh alone.”* However, midwives also said they tended to rely on the woman’s individual item responses and use those as a springboard for discussion. Another said, “*I don’t focus as much on a score, more the particular questions and what’s going on for the woman”.*

#### Sub theme 5.3 tailored action prompts

Overall, the PIPA action prompts were reported to be quite helpful when determining a woman’s referral pathway by both survey respondents (Table 4, Q3) and focus group participants. Compared to Usual Care, survey responses indicated that the action prompts in the PIPA model were more helpful when determining the woman’s referral needs than the Usual Care alerts (Table 2, Q11, 12). In the focus groups midwives commented, “*Yeah, I do [find the action prompts helpful], I don’t mind them at all.”* One midwife reported that she had not noticed the action prompts.

#### Sub theme 5.4: the clinician’s concerns box: the importance of clinical judgement

Overall, midwives’ comments on the summary information (action prompts, total score and risk level) suggested that they tended to rely on their clinical judgement based on responses to the individual questions rather than on the summary information. Midwives’ comments indicated a high level of comfort in using their professional judgement when formulating their view of a woman’s psychosocial challenges based on the psychosocial assessment, and when reviewing the EPDS and ANRQ-R scores.

The ‘clinician concerns’ free text box in the PIPA model enabled midwives to document their clinical judgement. One midwife commented, “*And being able to raise that … there [are] … clinician concerns. I think that’s really good, because... you might have an inkling [that there is more going on for a woman than her onscreen responses would suggest]”.*

The clinical concerns box was also used to document concerns where midwives disagreed with, or perceived discrepancies between, the computerised assessment results and the woman’s clinical presentation. Another midwife stated, “*And the other ones who get the low EPDS and low ANRQ-R, but they’re crying and upset. Then it’s good to be able to write that down [in the clinical concerns text box]. Because it allows you to … flag this and keep a close eye on this lady next time”.*

The free text box enabled the midwife to provide an explanation of a situation or context and to effectively “de-escalate” a score for example, “*Sometimes I’m putting something in the clinical concerns, which is “this looks like it is concerning”, but with some detail it’s not”.*

Midwives also commented that the details recorded in the clinician concerns text box and in the summary report were very useful because they and other midwives often worked with women that they had not seen at the booking visit but saw as their pregnancy progressed, saying that “*So I might be sitting in front of you but never met you before. So these summaries, for me who sees a lot of women are a … godsend”.*

Participants also emphasised the importance of the ‘clinical concerns’ and summary report for the MCD committee. One midwife said,

*I’m always thinking about that (MCD) meeting... Do they have the information they need to be able to make a judgment about this woman? Because if the score doesn’t actually reflect the woman … and I don’t actually think she’s of any concern, I’ll still … (flag her for the MCD meeting), but I’ll put it in my clinical assessment (clinician concerns text box) as well.*

## Discussion

Overall midwives who participated in the study were positive about psychosocial assessment and depression screening and comfortable asking women the questions. They expressed confidence in their skills and perceived they approached assessment in a sensitive way. Concern was expressed only in relation to addressing the more complex areas of DV disclosure and question 10 (self-harm) on the EPDS. This confidence likely reflects this sample’s level of midwifery experience both more broadly and with psychosocial assessment.

Midwives perceived psychosocial assessment had benefits for women. Offering the opportunity for women (‘opening the door’) to discuss their individual needs. Research in Australia [23] and Canada [24] supports the idea that women may not necessarily disclose their concerns immediately but may do so in an ongoing healthcare encounter. Despite routine depression screening being commonly reported as acceptable [25–27], recent research also indicates not all women disclose mental health concerns when asked [28]. In a large national study, Forder et al. found that one in five women stated they had not always responded to screening questions honestly and almost 40% indicated feeling some degree of discomfort when asked about their emotional wellbeing. Women less likely to respond honestly were those with a history of mental health issues, financial strain, low emotional/social support, and/or experiencing partner abuse [29]. Our midwife participants emphasised the need to gain trust by first explaining to women why they were asking these questions. In an ethnographic study of midwives’ practice, Rollans and colleagues also observed the importance of this and noted if explanations were not provided women were often perplexed about the questions [8, 30].

### The PIPA model compared to usual care

#### Asking the right questions

Making psychosocial assessment effective in a non-mental health setting where time is limited remains a challenge. Clear, simple wording of questions that are user friendly, both for woman and midwife, is important in helping to minimise misinterpretation, especially where a woman has limited command of English or a midwife is less experienced in psychosocial assessment [23]. The fact that midwives relied on the set wording of questions in the PIPA model more frequently than in Usual Care suggests they found this questionnaire (ANRQ-R) easier to administer. However, midwives will at times rephrase questions to aid interpretation particularly with women from culturally and linguistically diverse backgrounds. At times this rephrasing is appropriate and clarifies the question for the woman and at other times the reinterpretation is inappropriate [8]. Midwives also report that having to rephrase the question requires extra time, which is frustrating [23].

The ANRQ-R includes a question about the woman’s relationship with her mother when she was growing up. This question is informed by research on the intergenerational transmission of attachment difficulty and the view that adult attachment may predict child attachment [31]. In the focus group discussions, midwives emphasised the value of enquiring about women’s relationship with their mother and identifying women they believed would benefit from referral for attachment based counselling [31]. On the other hand, participants were concerned that the question related to women’s perception of their relationship with their partner (as asked in Usual Care) was absent. However, they did note that screening for domestic violence and enquiry about emotional support from their partner is included in the ANRQ-R.

#### Value of the PIPA risk score and summary reports in each model

The PIPA psychosocial risk summary report provided midwives with total EPDS and ANRQ-R total scores, level of psychosocial risk and which (if any) tailored referral pathway(s) were recommended. Many midwives reported the ANRQ-R total score and risk level of little use, preferring instead to review the detailed summary of psychosocial factors. This document, which is more detailed than the equivalent Usual Care summary document, was found to be particularly useful for midwives providing ongoing care where they had not personally undertaken the initial assessment. While not the focus of this paper, professionals who offer psychosocial support services have reported that they appreciate the detailed information that comes from a validated and routine screening process [23]. For example, in some refugee health services, staff appreciated the more comprehensive referral information as it added greater context to the woman’s situation at the time of triage and initial contact [23]. Similarly, the participants also emphasised the importance of summaries having all the information required by the MCD meeting to offer women the most appropriate services.

Some midwives reported that they would often refer a woman for psychosocial service discussion regardless of the risk level identified. This could be because the midwife’s formulation of the woman’s level of risk did not always match the programmed PIPA risk level and this issue is addressed in a separate paper (in submission). The use of clinical judgement to determine a woman’s risks and strengths is an important aspect of midwives’ work and relies on integrating information from many sources including a woman’s physical health and access to social support [32]. Midwives were thus particularly favourable to the ‘clinician concerns’ free text box in the PIPA folder which reflects this clinical process. Critically, the ‘clinician concerns’ section allows the clinician to identify a woman who is at risk based on clinical judgement even when no risk is detected through the pre-programmed questionnaire items alone.

#### Broader factors relating to psychosocial assessment

Both PIPA and Usual Care psychosocial question are computerised, incorporated into the electronic clinical information system used at the study site. Some midwives expressed frustration that they spent time interacting with the computer rather than engaging with the woman, a concern reported elsewhere in the literature [33]. To mitigate this, some midwives completed the psychosocial assessment on the computer together with the woman. Many midwives considered that the quality of information was better if jointly completed, enabling clarification of questions and responses as required, and facilitating rapport, particularly for highly sensitive or unexpected questions. In contrast other studies using digital screening indicate that there is a preference both by women [26] and clinicians [23] for screening to be conducted away from the health professional and then the results discussed with the woman.

Midwives reported that they felt well supported and were clear about referral pathways. The study site and others in NSW and Australia have invested in building effective referral pathways for women [23, 34–36]. These improvements are important because one of the commonly reported barriers to implementing routine screening is the limited referral pathways available. In their recent review, Viverious and Darling reported that midwives lacked knowledge regarding the referral processes involved in accessing perinatal mental health services or were unaware of the services altogether [37]. Without such support this type of program (whether Usual Care or PIPA) is likely to flounder.

Time was the one factor that was consistently reported as reducing midwives’ capacity to conduct the assessment optimally. While this was reported as more of an issue in the more comprehensive PIPA model, the implementation of the PIPA model coincided with the commencement of a new maternity computerised data collection system, eMaternity, which also included a broader, more time consuming series of intake questions outside of the psychosocial assessment. Having insufficient time to conduct assessments is commonly reported throughout the literature and there is a risk that in time pressured environments midwives may approach assessment as a check list [8, 33, 38] which can impede women’s ability to engage with their midwife [30, 33, 37, 39]. It is recommended that routinely-administered screening tools should be used as a means of facilitating conversation with clients, as opposed to form-filling [37]. Midwives in the present study were each finding their own way of to undertake a comprehensive, meaningful assessment within the time available to them.

### Implications for practice

Australia is a world leader implementing routine psychosocial assessment and depression screening into antenatal care, first introducing screening in 2000 [34]. Workplace culture can shift over time, with research conducted in NSW in 2008 [40]) and 2010 [8] demonstrating that some staff were uncomfortable with psychosocial screening; however, research conducted 5 to 8 years later demonstrated a greater level of comfort [23, 41]. This change was reflected in the findings of the present study, with midwives describing psychosocial screening as ‘second nature’, and demonstrates how over time what can be seen as challenging can become normalised and embedded in practice [38]. In other countries such as UK and Ireland where depression screening or broader psychosocial assessment is not currently routine, midwives report low levels of confidence in their ability to screen and assess women [42–44]. Noonan and colleagues, for example, reported that only 17.8% of midwives felt well equipped to support women [43].

Ongoing training and clinical supervision is critical to ensure that midwives feel prepared to conduct psychosocial assessment. Most midwives in the present study reported that they had not been offered regular training to support skill development though they did acknowledge brief training specifically related to the implementation of the PIPA model. Others report that across NSW there are infrequent training opportunities for midwives in psychosocial assessment [8, 40, 41, 45]. Thus our participants’ reported confidence in this domain may have been a result of workplace culture, practice and experience: the study site has provided consistent psychosocial and mental health support to women – including midwife-conducted screening - over the last 20 years.

Research exploring women’s experiences has highlighted that a lack of knowledge of perinatal mental health among health professionals can act as a barrier to women’s access to care [42, 46]. Training interventions in this area have been shown to be effective in improving knowledge and confidence across international settings [41].

### Study limitations

This study has a number of limitations. First, the total sample size of 44 midwives who completed the surveys, pre and or post PIPA implementation is a relatively small sample that would limit generalisation of the findings. In addition, around two thirds of midwives working in the antenatal clinic across the 2 year period and conducting psychosocial assessment completed the surveys in phase one and phase two. This may have introduced response bias and we were unable to gauge how representative participants were of the total group. In addition, only nine midwives completed the survey in both conditions and this meant that we were unable to undertake a within group pre and post comparison.

The participants in this study were generally very experienced, conducting psychosocial assessment and depression screening for an average of seven and a half years and 9 years at each time point. Only two midwives had fewer than 5 years’ clinical experience. They also had psychosocial support services available to them within the hospital (including mental health nursing, perinatal psychiatry, social work and psychologists). Hence, results may not be generalisable to settings where midwives have less experience and/or access to fewer options for onward referral. We only conducted two focus groups with midwives with a total of 16 participants. The midwives all knew each other and this may have limited participant disclosure on any concerns they had as individuals in undertaking assessment and screening. However, we would suggest that all, or the vast majority, of midwives felt comfortable to speak freely – as evidenced by the variety of views expressed, including a number of contradictory viewpoints.

## Conclusion

This mixed methods study aimed to describe midwives’ perspectives on psychosocial assessment and depression screening at the antenatal booking appointment and to compare their perspectives and experiences on the two models – Usual Care versus the PIPA model. Overall, midwives were positive about psychosocial assessment and depression screening, indicating that there were benefits for women and that this assessment assisted them to gain a greater understanding of women’s social and emotional needs. The participants were comfortable asking women the questions and highlighted the importance of conducting screening and assessment in a sensitive way, adapting their approach accordingly. The PIPA model facilitated improvements in three areas: first, greater reliance by midwives on the suggested wording of the psychosocial questions; second, the referral or action messages displayed at the end of the PIPA psychosocial assessment were more helpful than those in Usual Care and third, around 70% of midwives indicated they were more likely to follow these messages than the generic alert in the Usual Ccare. Midwives also valued the opportunity to express their clinical judgement by using the space provided in the clinician concerns free text box. Given the overall lack of training and experience undertaking psychosocial assessment reported by midwives in other maternity settings nationally and internationally, the PIPA model with its more structured and detailed questionnaire (ANRQ-R) and referral prompts may be better adapted to implementing in less resourced settings.

## Supplementary information

**Additional file 1.** Comparison of key features of the SAFE START and PIPA models of integrated psychosocial care.

**Additional file 2.** Midwives focus group questions.

## Data Availability

The datasets generated and/or analysed during the current study are not publicly available due to the terms of the study’s ethical approval. Access to non-identifiable study data can be provided to third parties only with permission of the Principal Investigator and following specific ethical approval.

## Abbreviations

ANRQ-R: Antenatal Risk Questionnaire-Revised
EPDS: Edinburgh Postnatal Depression Scale
GP: General Practitioner
MGP: Midwifery Group Practice
MCD: Multidisciplinary Case Discussion
NSW: New South Wales
PIPA: Perinatal Integrated Psychosocial Assessment
QUAL: Qualitative arm of the study
QUANT: Quantitative arm of the study
SPSS: Statistical Package for the Social Sciences
UK: United Kingdom
