# Supplementary material for: Opening the door: midwives’ perceptions of two models of psychosocial assessment in pregnancy- a mixed methods study
Source: BMC Pregnancy Childbirth. 2020 Aug 7;20:451. doi: 10.1186/s12884-020-03133-1 (PMC7412833; doi:10.1186/s12884-020-03133-1)
Supplement: Supplementary file 1 — Additional file 1. Comparison of key features of the SAFE START and PIPA models of integrated psychosocial care. [file 12884_2020_3133_MOESM1_ESM.docx]

Supplementary file 1: Comparison of key features of the SAFE START and PIPA models of integrated psychosocial care(1)

|  | **Model of integrated psychosocial care** | |
| --- | --- | --- |
|  | **SAFE START model**  **(Care as usual)** | **PIPA model**  **(alternative model)** |
| **Psychosocial assessment measures** | EPDS, SAFE START psychosocial questions | EPDS, ANRQ-R (psychosocial questions); clinician concerns |
| **Psychosocial risk levels** | ***Three levels of psychosocial risk, defined as:***  ***Level 1***: no specific vulnerabilities or risk  ***Level 2***: *one or more* *risk factors* of variable severity and significance including, but not limited to, low supports, multiple birth, financial stress, isolation, ‘mild-moderate’ depression or anxiety, history of mental health problem, young age.  ***Level 3***: *one or more of four complex risk factors* (domestic violence, involvement with child protection services, substance misuse, severe mental illness) | ***Six levels of psychosocial risk, defined as:***  ***No risk*:** no risk factors endorsed on ANRQ-R; EPDS < 13 (Q10=0); no clinician concerns.  ***No risk on ANRQ-R but clinician concerns and/or EPDS = 13 or 14.***  ***Low risk*:** presence of anxious or perfectionistic personality style, mental health history without functional impact or any one of low social or partner support, or recent significant stressors **and** EPDS < 15 (Q10=0).  ***Medium risk*:** Childhood trauma **or** neglect **or** 2-4 of low social or partner support, recent significant stressors or young age **and** EPDS < 15 (Q10=0).  ***Medium-high risk*:** ANRQ-R ≥25 (excluding any ‘complex’ risk factors^a^ or combination of ‘social’ risk factors^b^) **or** EPDS ≥15 (Q10 =0) **or** childhood trauma **and** neglect.  ***High risk*:** ANRQ-R >25 **and** other ‘social’ risk factors^b^ **or** any ‘complex’ risk factor(s) ^c^ or EPDS Q10>1. |
| **MCD meeting referral threshold** | Levels 2 and 3 | Medium Risk Level upwards **or** suicidal or self-harm ideation (irrespective of total EPDS score or ANRQ-R responses). |

MCD: multidisciplinary case discussion meeting; ANRQ-R: Antenatal Risk Questionnaire-Revised; EPDS: Edinburgh Postnatal Depression Scale.

^a^ ‘Social’ risk factors: young maternal age (less than 20 years); no partner; booking-in appointment at >20 weeks gestation;

^b^ ‘Complex’ risk factors : homelessness or housing instability; domestic violence; substance misuse; contact with child protection services.

1. Reilly N, Black E, Chambers GM, Schmied V, Matthey S, Farrell J, et al. Study protocol for a comparative effectiveness trial of two models of perinatal integrated psychosocial assessment: The PIPA project. BMC Pregnancy and Childbirth. 2017;17(1).
