# Supplementary material for: Opening the door: midwives’ perceptions of two models of psychosocial assessment in pregnancy- a mixed methods study
Source: BMC Pregnancy Childbirth. 2020 Aug 7;20:451. doi: 10.1186/s12884-020-03133-1 (PMC7412833; doi:10.1186/s12884-020-03133-1)
Supplement: Supplementary file 2 — Additional file 2. Midwives focus group questions. [file 12884_2020_3133_MOESM2_ESM.docx]

**SUPPLEMENTARY FILE 2 Midwives focus group questions**

**Preamble:** Psychosocial assessment and depression screening have been undertaken for around 15 years at this hospital. More recently this assessment has gone from SAFE START in ObstetriX to the PIPA model embedded in e-maternity.

Today we want to hear about your experiences with the PIPA model embedded in e-maternity **in terms of psychosocial assessment** and compare it to your experiences using SAFE START embedded in ObstetriX. We also want to know about your experiences of **psychosocial assessment more generally**.

****PLEASE REMIND GROUP THAT RESPONSES WILL BE DE-IDENTIFIED****

**INTRODUCTORY QUESTIONS**

1. In general, describe your experiences undertaking psychosocial assessment and depression screening?
2. What do you think are the positive aspects of PIPA **for women**?
3. What do you think are the negative aspects of PIPA **for women**?
4. In general, how do you prepare women for psychosocial assessment?
   1. Prompt this might include – room set up or how you use the computer)
5. Thinking about how you facilitate psychosocial assessments, what factors influence whether the women
   1. Self-completes all/part of the assessment
   2. Completes the assessment sitting next to you (working through the questions together)
   3. Answers questions read out loud by you
6. What factors facilitate the psychosocial assessment process?
7. What factors hinder the psychosocial assessment process?
8. **Thinking about key differences between Obstetrix and PIPA JUST** in terms of the **psychosocial assessment** – how have the changes impacted on your experiences?

Prompts

- 1. Availability of a total score for the psychosocial assessment (ANRQ), as well as the EPDS
  2. EPDS Q10 self-harm exploration questions (has anyone used these yet?)
  3. Assignment of a level of psychosocial risk e.g. high, medium, low, no risk,
  4. Action prompts relating to the level of psychosocial risk
  5. Serious clinicians concern free text box
  6. Structure and content of the PIPA psychosocial summary report

1. **For you as a midwife:**
   1. Has your practice changed since PIPA has been in place? If so, how?
   2. What are the positive and negative aspects of PIPA?
2. What is your experience of the referral process, e.g. once you put the file into the black box?
